# Premature subclinical atherosclerosis in children and young adults with juvenile idiopathic arthritis. A review considering preventive measures

**DOI:** 10.1186/s12969-015-0061-5

**Published:** 2016-01-06

**Authors:** Anna-Helene Bohr, Robert C. Fuhlbrigge, Freddy Karup Pedersen, Sarah D. de Ferranti, Klaus Müller

**Affiliations:** Department of Paediatrics and Adolescent Medicine, JMC Research Unit, Rigshospitalet Afs. 7821, Tagensvej 22, DK 2200 Copenhagen N, Denmark; Children’s Hospital Boston, Boston, USA; Department of Paediatrics and Adolescent Medicine, Rigshospitalet, Copenhagen, Denmark; Department of Paediatrics and Adolescent Medicine, and Institute for Inflammation Research, Rigshospitalet, Copenhagen, Denmark

**Keywords:** Juvenile idiopathic arthritis, Chronic arthritis, Premature atherosclerosis, Adipositas, BMI, Physical activity, Tobacco smoking, Prevention of atherosclerosis

## Abstract

Many studies show that Juvenile Idiopathic Arthritis (JIA) is associated with early subclinical signs of atherosclerosis. Chronic inflammation *per se* may be an important driver but other known risk factors, such as dyslipidemia, hypertension, insulin insensitivity, a physically inactive lifestyle, obesity, and tobacco smoking may also contribute substantially. We performed a systematic review of studies through the last 20 years on early signs of subclinical atherosclerosis in children and adolescents with JIA with the purpose of investigating whether possible risk factors, other than inflammation, were considered.

We found 13 descriptive cross sectional studies with healthy controls, one intervention study and two studies on adults diagnosed with JIA. Only one study addressed obesity, and physical activity (PA) has only been assessed in one study on adults with JIA and only by self-reporting. This is important as studies on PA in children with JIA have shown that most patients are less physically active than their healthy peers, and as physical inactivity in several large studies of normal schoolchildren is found to be associated with increased clustering of risk factors for cardiovascular disease. It is thus possible that an inactive lifestyle in patients with JIA is an important contributor to development of the subclinical signs of atherosclerosis seen in children with JIA, and that promotion of an active lifestyle in childhood and adolescence may diminish the risk for premature atherosclerotic events in adulthood.

## Background

Juvenile Idiopathic Arthritis (JIA) is the collective term for a clinically diverse group of rheumatic inflammatory syndromes of unknown etiology, which may present as a systemic inflammation, an isolated arthritis or in association with other organ specific inflammatory disorders such as psoriasis and uveitis. The annual incidence in the Western world is 16–150 per 100.000 children, making it the most common chronic inflammatory disease in childhood [1]. Several phenotypes are recognized, ranging from self-limiting forms involving a few joints, to erosive polyarthritis and systemic JIA (sJIA), all of a relapsing and remitting nature. Although some patients enter spontaneous permanent remission, 41–78 % of patients require continuous or recurrent treatment in adulthood [1–4]. JIA is thus a long lasting chronic inflammatory disease, and concern has been raised, as in rheumatoid arthritis (RA), regarding the risk of premature development of cardiovascular disease. Indeed, several studies of children with JIA have described the occurrence of early subclinical signs of atherosclerosis.

Family disposition for cardiovascular disease, dyslipidemia, hypertension, and diabetes, as well as lifestyle factors such as obesity, physical inactivity, and tobacco smoking are known individually significant risk factors for accelerated development of atherosclerosis; cohort studies of the general population such as Framingham Heart Study [5] and Young Finns [6, 7] have helped in identifying these factors. More recently the concept has emerged that chronic systemic inflammation may also contribute. This is based primarily on large cohort studies of patients with RA. For example, a meta-analysis of mainly community based cohorts and case–control studies of incident cardiovascular events including myocardial infarction, cerebrovascular accidents and congestive heart failure in 41,490 patients with RA indicates an increase in risk of about fifty percent compared with the general population [8]. Likewise treating to the lowest possible levels of disease activity has been shown to reduce the risk for cardiovascular events in patients with RA [9].

However, focusing too narrowly on persistent inflammation as a driver for the development of atherosclerosis risks overlooking equally important, and potentially reversible, risk factors; this is well-illustrated in a recent report on progression of atherosclerosis in patients with RA by del Rincón et al. [10]. For this reason we reviewed existing studies reporting early or subclinical signs of cardiovascular disease in children with JIA to see whether known risk factors were taken into account.

Studies published in English were searched through PubMed (National Library of Medicine), primarily making use of MeSH terms and free text and secondarily by following key references in relevant articles. Studies from the last 20 years are included, as this covers a period of more efficacious treatment for JIA.

### Risk for development of premature atherosclerosis in childhood and adolescence

Acquired overt cardiovascular disease is rare during childhood. However, *post mortem* studies of the vasculature of apparently healthy children and young adults, 2 to 39 years of age, have shown that microscopic lipid deposits and inflammatory reactions, the hallmark of atherosclerosis, are found in the arterial intima of infants and young children and that fatty streaks and fibrous plaques are seen in the aorta and coronary arteries of most teen-agers [11–14], suggestive of atherosclerosis as a continuing process beginning early in life. The natural history of the arterial lesions was investigated through studies at the same location in the arterial tree across different age groups. Progression to more severe atherosclerosis was associated with raised levels of the non-High-Density-Lipoprotein fraction of cholesterol (non-cHDL) in blood, hypertension, impaired glucose tolerance, obesity, and tobacco smoking, with each factor reinforcing the others [15].

In addition there is mounting evidence for the importance of physical activity (PA) for continuing cardiovascular health through childhood, adolescence and adulthood [16–21]. Indeed, being physically active is one of the seven ideal metrics for continuing cardiovascular health issued by the American Heart Association [22], the other six metrics being non-smoking, keeping a healthy diet, maintaining normal blood pressure, normal glucose- and lipid-metabolism, and normal weight.

Large longitudinal observational population-based studies beginning in childhood or adolescence confirm the association with structural or functional vascular changes in adulthood, indicative of future clinically important cardiovascular disease [6, 23–28] (Table 1).

**Table 1:** Known risk factors in childhood and adolescence for premature development of cardiovascular disease

Family disposition	
Hypertension	
Hypercholesterolemia, dyslipidemia	
Insulin resistance	
Obesity	
Physical inactivity	
Smoking	

References given in the text

### Surrogate markers of preclinical atherosclerosis

In adults, several non-invasive techniques for evaluation of endothelial function and structural changes in the arterial wall have proven reliable markers for later development of acute cardiovascular events and are now included in many clinical studies as surrogate markers of atherosclerosis.

In a Scientific Statement from the American Heart Association, Urbina et al. review assessment of subclinical atherosclerosis in children and adolescents by these techniques [29].

A short description of the non-invasive methods used in investigations of cardiovascular function in JIA is given in Table 2, together with relevant references.

**Table 2:** Non-invasive methods for investigation of cardiovascular function

Measurement	Abbreviation	Principle	References
Coronary artery calcification	CAC	Arterial wall structure, atherosclerotic plaques	[7, 29, 37]
Intimal and Medial thickness of the wall in carotis or aorta	cIMT (carotis) , aIMT (aorta)	Arterial wall structure	[21, 26, 29, 71]
Left ventricle mass index	LVMi	Left ventricular dimensions adjusted for height, weight, age, and sex	[32]
Pulse wave velocity	PWV	Direct measure of stiffness in large arteries	[29, 37, 72, 73]
Augmentation index	AIx	Indirect measure of arterial stiffness combining arterial and ventricular function	[29, 37, 73]
Flow mediated dilation	FMD	Endothelial cell function	[29, 71, 74–76]
Glyceryl trinitrate mediated flow	GTN-mediated dilation	Arterial wall function	[71]
Arterial distensibility		Direct measure of stiffness in large arteries	[29, 71]
Plasma natriuretic peptide	NT-pro-BNP	Ventricular dysfunction	[77]
Troponin T	TnT	Myocardial damage	[77]

As well-defined atherosclerotic events like myocardial infarcts and stroke are very rare in childhood, the prognostic value of the described abnormalities must await clinical studies reaching into mid-late adulthood. Nevertheless the association established between the structural and functional surrogate markers of early atherosclerosis and the above-mentioned known risk factors for development of clinical overt atherosclerosis [6] make these simple and non-invasive techniques attractive as tools in studies of cardiovascular health in children with JIA.

At present, there are no available prognostic biomarkers in the blood with acceptable sensitivity and specificity for subclinical cardiovascular disease [30, 31].

### Investigations of cardiovascular structure and function in children and adolescents with JIA

Although severe extra-articular complications may occur in the acute phase of systemic JIA (sJIA), including serositis, myocarditis, renal amyloidosis, and cerebral vasculitis, there is no evidence of cardiac and cardiovascular involvement as common clinical features in the chronic phase of JIA during childhood and adolescence. However, JIA is a chronic inflammatory disease, and concern regarding premature development of cardiovascular disease, as seen in patients with RA, has led to performance of echocardiographic and tonometric studies, as well as studies on endothelial function in children and young adults with JIA with no clinical signs of cardiovascular dysfunction and with no family disposition for cardiovascular disease (Table 3).

**Table 3:** Investigations of structure and function of heart and / or arteries in children and adolescents with JIA with no clinical signs of cardiovascular dysfunction

Ref.	Design	No. of patients and controls	Age-group	Numbers of patients and subtypes	Number of patients in treatment at time of investigation	Study parameters	Significant findings
Stamato et al. 1995 [78]	Descriptive cross-sectional	36	10–17.5	36 HLA-B27 pos. with spondylarthropathy	No information	Echocardiographic assessment of left ventricle and the outflow tract.	Mild aortal regurgitation in patients unrelated to disease duration
	with an age matched healthy control group	33 ^*^	6-18 ^*^			Atrio-ventricular conduction	
						Disease duration	
Huppertz et al. 2000 [79]	Descriptive cross- sectional	40	6–26	35 HLA-B27 pos ERA	No information	Echocardiographic assessment of the left ventricle functions before and after exercise.	HLA-B27 positive ERA possibly at risk for development of aortic regurgitation and impaired myocardial relaxation
	with a control group of age and sexmatched HLA-B27 neg JIA and 25 healthy children	15 + 25 ^*^	6 - 25 ^*^	3 oligo		Atrio-ventricular conduction	
				1 sJIA		BP	
				1 unclassified			
Oguz et al. 2000 [80]	Descriptive cross- sectional.	30	3–15	19 oligo	Mainly NSAID	Echocardiographic assessment of the left ventricle function	Higher systolic and diastolic BP, but within normal limits, and diastolic dysfunction of abnormal relaxation type in patients
	with an age matched healthy control group	30 ^*^		10 poly	The patient with systemic JIA received corticosteroid	BP	
				1 sJIA.	One unspecified patient received MTX		
Argyropoulou et al. 2003 [81]	Descriptive cross-sectional	31	No data	18 oligo	No information	Evaluation by MR of aortic distensibility and PWV	Lower distensibility and higher PWV in patients unrelated to JIA subtype
	with an age matched healthy control group	28 ^*^		6 poly		Disease activity	No correlations between aortic distensibility / PWV and metabolic and disease activity parameters
						Insulin sensitivity	
						Lipid profile	
				7 sJIA			
Bharti et al. 2004 [82]	Descriptive cross-sectional.	35	No data	oligo	All received NSAID	Eccocardiographic evaluation of left ventricular function	Higher systolic and diastolic BP, but within normal rate, and higher resting heart rate in patients.
	with an age matched healthy control group	35 ^*^		poly			Diastolic dysfunction and higher systolic and diastolic dimensions and volumes.
				sJIA			
				No numbers given			
Pietrewicz et al. 2007 [83]	Descriptive cross-sectional	40	4–16	32 oligo	No information	Echocardiographic assessment of cIMT	Increased cIMT in patients with JIA, highest in children with polyarthritis, and correlation between homocystein and cIMT
						Homocysteine	
	with an age matched control group of healthy children	23 ^*^	3–17 ^*^	8 poly		CRP	
						Lipid profile	Correlation between disease duration and cIMT
						Disease duration	
Vlahos et al. 2011 [84]	Descriptive cross-sectional	30	7–18	15 oligo	3 NSAID	Echocardiographic assessment of cIMT	Reduced FMD in patients (as a group) associated with ESR but without any association to medication or clinical disease activity
	with a BMI, sex, and age matched control group of healthy children	33 ^*^		8 poly	4 corticosteroid	PWV	
						FMD	
				7 sJIA	15 MTX	Arterial compliance	Increased cIMT in sJIA compared to controls or non-systemic JIA and related to use of corticosteroids, disease activity, BMI, blood pressure, dyslipidaemia, and age
					9 TNF-inhibitor	Disease activity	
						BMI	
						BP	
						Glucose	
						Lipid profile	No difference in PWV or arterial compliance between groups
						Smoking	
Koca et al. 2012 [85]	Descriptive cross-sectional	50	5–16	22 oligo	No information	Echocardiographic assessment of left ventricle function	Impaired diastolic function in patients
				13 poly		Electrographic assessment	
							No arrhythmias
				6 ERA			
				4 PsA			
				5 sJIA			
	with a sex, and age matched control group of healthy children	70 ^*^					
	Follow-up after 12 month.						
Abul et al. 2012 [86]	Descriptive cross-sectional	55	12.57 SD 2.9	24 oligo	22 NSAID	Echocardiographic assessment of right ventricular function	Systolic and diastolic dysfunction of the right ventricle
				8 poly	31 Salazopyrin		
				15 ERA	31 MTX		
	with a BMI, sex, and age matched control group of healthy children	33 ^*^	11.9 SD 2.7 ^*^	1 PsA	25 Corticosteroid	Disease activity	No association to medication including steroids and no associations to disease activity
				7 sJIA	2 TNF-inhibitor		
Alkady et al. 2012 [66]	Descriptive cross- sectional	45	5–16	5 oligo	NSAID	Echocardiographic assessment of systolic and diastolic function (36 patients)	Higher resting heart rate and higher systolic and diastolic BP in patients but within normal range. Also enlarged left ventricular systolic dimensions and diastolic dysfunction. In 6 patients was found thickened pericardium, and in 9 mitral valve thickening and mild dysfunction.No association with disease activity reported.
				10 poly	26 MTX		
				20 ERA	8 Corticosteroid		
	with a sex and age matched control group of healthy children	30 ^*^		1 PsA		Spirometry and CO diffusion (30 patients)	
				9 sJIA			
						23 patients and controls had both investigations	
						Disease activity and duration	
							In 19 out of 30 patients was found a reduction in pulmonary function primarily of a restrictive pattern, inversely correlated to disease duration and severity / treatment with MTX
Breda et al. 2012 and 2013 [33, 34]	Longitudinal intervention study of 12 months	38	4.7–9.4	Oligo- or poly	NSAID	cIMT	Improvement in all baseline disease parameters, including BT, after one year of “ treatment to target” except cHDL that was found normal at baseline and did not change. Positive correlation between cIMT and LDL and IL-1beta, no correlation to CRP or ESR. BT was found elevated at baseline but within normal range
				Mild disease in 22	MTX at baseline.	Clinical disease activity	
						ESR, CRP	
	with a sex, age and puberty stage matched control group of healthy children	40 ^*^	4.1- 8.6^*^	Aggressive disease in 16 with poly	During follow-up disease control was obtained by 22 in treatment with NSAID +/- conventional DMARDs	Proinflammatory cytokines	
						BP	
						Lipid profile	
						Oxidant status	
					16 patients needed more aggressive treatment with TNF-alfa inhibition		
Glowinska-Olszewska et al. 2013 [32]	Descriptive cross- sectional	58	11–15	28 oligo	42 Corticosteroid	BMI	22% of the patients met the criteria for overweight or obesity.
				26 poly	28 MTX	FMD	
				4 sJIA	14 Biologics	cIMT	
				Clin. active inflammation: 30	9 Unspec. DMARDS	LVMi	Lower FMD and higher cIMT, LVMi, BMI, and BP in patients as a group compared to controls; highest cIMT and lowest FMD in obese patients. No difference between patients with clinically active and inactive disease and no difference between JIA subtypes.
						Disease activity	
						BP	
						CRP	
						IL-6, TNF-alfa	
						Lipid profile	
						Insulin sensitivity	
	with a sex and age matched control group of healthy children with normal weight; no obese children	36 ^*^	12-15 ^*^	Clin. inactive inflammation: 28			
Raab et al. 2013 [36]	Descriptive cross- sectional study of young adults with severe JIA, based on self-reports	344	19.7 SD 2.8	28 oligo	215 Biologics	Comorbidity	In 9.9% were reported CVD with hypertension in 7.3%, not different from the control group
				50 extended oligo			
				91 RFneg poly			
				37 RFpos poly	151 MTX	Disease activity	
				75 ERA	64 Other conventional	Health	CVD, mainly hypertension, was reported in 40.6% of 15 patients with sJIA
				37 PsA	DMARDs	Functional deficits,	
				15 sJIA			
				11 other arthritis			
	and compared to an age and sex matched cohort sampled from the general population	688 ^*^					
Aulie et al. 2014 [37]	Cross-sectional, observational study of patients with disease duration of more than 23 years	87	34.8–40.6	15 oligo	25 TNF-inhibitor	BP	Higher systolic and diastolic BT and small elevation of PWV in patients related to diastolic BT
				14 extended oligo	19 Methotrexate	PWV	
				13 RF neg poly	23 Daily NSAID	AIx	
				5 RF pos poly	6 Prednisolone	Coronary calcification	
				18 ERA		Disease activity	No difference in AIx between patients and controls, but a positive association to diastolic BP, accumulated disease parameters inclusive treatment with prednisolone, and daily smoking, and a negative association to vigorous physical activity
				15 PsA			
						CRP, ESR	
						BMI and waist circumference	
				4 sJIA		Lipid profile	
				3 unclassified		Insulin resistance	
						Self reported habits of smoking and physical activity	
	With an age and sex matched group without DM or inflammatory arthritis selected from a national population register	87 ^*^					
							Coronary calcification was present in 26% of patients, a frequency not different from that found in a large population study, and related to waist circumference, BMI, systolic BP, blood glucose and years on daily prednisolone
Lianza et al. 2014 [77]	Two year prospective observational study	21	2.2–17.8	21 poly	TNF-inhibitor	Systolic and diastolic cardiac function evaluated by echocardiography	Mild ventricular diastolic dysfunction in JIA with no relation to NT-pro-BNP. Possible association between NT-pro-BNP and disease activity.
	with age and sex matched healthy controls	22 ^*^	6 - 17 ^*^			Cardiac biomarkers: NT-pro-BNP	
						Troponin T	No sign of cardiovascular deterioration during treatment with TNF-alfa inhibitor.
						Disease activity	
Satija et al. 2014 [71]	Cross sectional, observational	31	3.5–16	2 oligo	No DMARD or biologics	cIMT,	Reduced arterial elasticity in patients indicative of increased stiffness, all had normal BT. No difference in cIMT, FMD, GTN-MD between subgroups and controls
				2 RF neg poly		Arterial elasticity FMD	
		31 ^*^					
						GTN-MD	
						BT	
				4 RF pos poly		Disease activity	
				9 ERA		ESR	
				14 sJIA		Lipid-profile	Correlation between cIMT and ESR
	With an age and sex matched control group of healthy children						

SD is given in brackets. *Aix* Augmentation index, *aIMT* aorta intima-media thickness, *BP* blood pressure, *CAC*, coronary artery calcification, *cIMT*, carotis intima-media thickness, *ERA* Entesitis-related arthritis, *ESR* erythrocyte sedimentation rate, *FMD* flow mediated dilatation, *GTN*-*MD* glyceryl trinitrate mediated dilatation, *LVMi* left ventricle mass index, *MTX* Methotrexate, *NSAID* Non Steroid Anti-Inflammatory Drug, *Oligo* oligoarticular JIA, *RF* Rheuma-factor, *Poly* Polyarticular JIA, *PsA* Psoriasis associated JIA, *sJIA* systemic JIA, *DMARD* disease modifying anti-rheumatic drugs, *PWV* pulse wave velocity

In the available cross-sectional studies measuring signs of early atherosclerosis in JIA, life style risk factors for development of premature atherosclerosis were not, in general, considered systematically. Lipids were measured in several studies and showed no consistent pattern, but only one study specifically addressed overweight status [32]. None of the studies took PA into consideration.

There is, at present, only one intervention study [33, 34] that has examined the effect of anti-inflammatory treatment on cIMT. In a group of prepubertal patients with oligo- and polyarticular JIA, with a control group only at baseline, cIMT was found to be significantly increased in JIA patients at enrolment, with a significant decrease documented after 1 year of anti-inflammatory medication, (NSAID, MTX, Etanercept). Treatment was also correlated with a significant reduction in diastolic and systolic blood pressure and an improvement in inflammatory markers and lipids. Lifestyle was not documented, however, leaving open the possibility that the cardiovascular improvement was due to a healthier, more active lifestyle which might have occurred in parallel with decreasing disease activity.

Prospective long-term studies of JIA have focused on the prevalence and severity of arthritis and the impact on musculoskeletal function; only few studies report data on cardiovascular health in adults with a history of JIA [35–37]. Raab et al. [36] collected information from adult patients with JIA treated with biologics. Cardiovascular disease, mainly arterial hypertension, was reported in a total of 9.9 %, a proportion similar to that seen in an age and gender matched control group drawn from a community sample. However, a disproportionally high rate was noted in patients with a history of sJIA, where 6 out of 15 patients reported hypertension; 2/3 of the patients with sJIA received treatment with corticosteroids.

Aulie et al. [37] examined arterial stiffness by use of PWV and AIx in a 29-year follow up study of young adults diagnosed with JIA and still having active disease. These authors found a small, but significant, increase in arterial stiffness by PWV associated with elevated diastolic blood pressure. AIx was not significantly different from controls but was negatively correlated with markers of active disease, use of prednisolone, self-reported lower PA, and daily smoking. Coronary artery calcification was also not more frequent in young adults with JIA than in the general population, but was positively correlated with waist circumference, BMI, systolic blood pressure, blood glucose and daily prednisolone. Insulin resistance was increased in the patients as was, unexpectedly, the frequency of daily smoking. The study by Aulie et al. [37] is the only report in which all known risk factors for atherosclerosis were taken into consideration along with disease characteristics. Assessment of PA, however, was only documented by self-report and not objectively measured.

### Risk factors for premature subclinical atherosclerosis in JIA

The studies included in this review use various techniques for the assessment of cardiovascular function and, except for the studies of young adults with JIA, include relatively small groups of children or adolescents with variable subtypes of JIA in diverse states of activity and on different medications. These different circumstances make meta- and subgroup-analyses difficult. Nevertheless, taken together we find it reasonable to conclude that surrogate markers of early atherosclerosis are present more often in JIA patients than in their healthy peers. Several studies, including those of young adults with JIA, show a significantly higher rate of elevated blood pressure, ventricular dysfunction and increased cIMT as a general feature of JIA, possibly associated with more pronounced systemic inflammation and dyslipidemia. Also the signs of aortitis and myocarditis, seen most prominently in patients with ERA (Enthesitis-related arthritis), support the concept of persistent systemic inflammation as an important driver of premature cardiovascular disease.

Longer term follow-up studies, however, have not shown any increase in clinically overt atherosclerotic events in young adult (less than 41 years of age) with long-standing JIA, which points to a slowly-developing, multifaceted process which may be amenable to preventive measures.

Elevated blood pressure, prehypertension as seen in several of the studies, is associated with increased cIMT and Left Ventricle Mass Index (LVMi), diastolic dysfunction and arterial stiffness, independently of BMI but associated with dyslipidemia [38, 39]. Dyslipidemia may be a feature of persistent inflammation [40–42] and may be associated with Metabolic Syndrome (MetS), a cluster of independent risk factors for atherosclerosis [43] that are also associated with persistent inflammation [44]. The occurrence of MetS in JIA has not yet been studied in great detail (for a recent review see Zanette et al. [45]). Glucocorticoid treatment may lead to insulin resistance [46–49], which is a hallmark of MetS. Since the introduction of MTX and specific biological inhibitors of inflammation, glucocorticoids are typically used in lower dosages and for shorter periods of time, but may still be a concern regarding metabolic dysfunction, risk of hypertension and premature atherosclerosis as seen in the studies by Raab et al. [36] and Aulie et al. [37]. In European studies from 1969 and 1977, secondary amyloidosis, with risk of hypertension due to kidney deposition, was reported to occur in 5–7 % of JIA patients, most often in children with systemic JIA. In a retrospective hospital-based study in Turkey [50] looking at 196 children with JIA from 1995 to 2004, only three patients (1.4 %) developed amyloidosis a frequency comparable to that reported by Raab et al. [36]. Interestingly, information on secondary amyloidosis in children with JIA has only appeared in scattered case reports in the last few years; presumably the prevalence of secondary amyloidosis is declining, as more efficient anti-inflammatory medications have become available.

By addressing chronic inflammation aggressively, the impact of the known risk factors for premature development of atherosclerosis (i.e. hypertension, dyslipidemia, and insulin resistance), may well diminish in parallel with the decreasing inflammation; preventable risk factors (including overweight, physical inactivity, and tobacco smoking) should then be considered.

High BMI (overweight and obesity) is by itself, associated with low grade systemic inflammation [51–55]. As elevated BMI could, thus, potentially amplify a preexisting inflammatory condition and thereby enhance the risk of premature atherosclerosis, a number of studies have looked at overweight and obesity in patients with JIA. In a recent cross-sectional study of 154 American children and adolescents with JIA, 18 % met criteria for obesity and an additional 12 % were overweight, similarly to what is seen in otherwise healthy American children [56]. The authors did not find an association between obesity and clinical disease activity, duration of illness or medication; markers of inflammation (CRP and ESR) did not correlate with BMI. Statistical power was, however, limited in this relatively small study which did not include healthy controls. Two other small recent cross-sectional studies of children and adolescents with JIA, in Morocco [57] and Poland [32], found higher rates of obesity and overweight than reported in the national references. In the study from Poland obese patients had higher levels of inflammatory markers in the blood, dyslipidemia and signs of insulin resistance as well as higher blood pressure compared to normal weight patients, but there were no obese healthy children in the control group. A controlled cross-sectional study from Brazil, looking at body composition in 42 female children and adolescents with JIA, showed increased body fat and truncal fat in prepubertal children with JIA, independent of subtype and medication [58]; this finding is of interest since abdominal fat is considered the origin of systemic inflammation associated with obesity. Weight gain and increase in visceral fat have been described in patients with RA receiving TNF-α- and IL-6-inhibitors, but weight gain was not found in a cohort of children with JIA on TNF-α inhibitor therapy compared with JIA patients not treated with TNF-α inhibitors [59]; body composition was, however, not assessed.

Physical inactivity is another modifiable risk factor for premature atherosclerosis [16–21]. More specifically, a study on adolescents and young adults by Edwards et al. [18] showed that higher PA was an independent predictor for lower arterial stiffness, measured as peripheral arterial distensibility and AIx.

In children with JIA, a sedentary lifestyle due to pain, fatigue and sleep disturbances is not uncommon [60]. Lelieveld et al. [61] found low fitness and low levels of PA in adolescents with JIA compared to healthy children. The inactive lifestyle was, however, unrelated to the degree of disease activity, and remission of clinical symptoms did not result in a more active lifestyle, which signifies complex reasons for physical inactivity in these patients. Also a recent study [62] reported reduced PA unrelated to pain or objective signs of inflammation in children and adolescents with JIA. Unfortunately PA was only assessed in one of the investigations on subclinical atherosclerosis in JIA [37] and in that study only by self-reports, a less reliable means of assessment.

A cross-sectional study of fitness in children with JIA showed significantly lower aerobic capacity among children aged 6 to 11 years with polyarticular JIA compared with matched healthy controls [63]. This finding was supported by a subsequent meta-analysis of 5 studies with a total of 144 children [64] and a more recent study [65], in which the investigators found a significant negative correlation between disease activity and aerobic capacity in children, adolescents and young adults across all JIA subtypes. Also patients in remission were found to have reduced aerobic capacity. Details regarding PA are not given, but the authors state that the lower aerobic capacity was not simply explained by sedentary lifestyle, and the authors speculate that muscle wasting, lung dysfunction, as also found by Alkady et al. [66], and anemia due to chronic inflammation may be important contributing factors. In healthy children, Dencker et al. [67] also found only a weak association between PA and aerobic fitness. This could be due to assessment methods, as intervention studies show increased aerobic fitness connected with increased PA [17]. Muscle wasting, lung dysfunction, and anemia due to chronic inflammation should diminish in the wake of more effective disease control, thus making regular PA possible for patients with JIA.

Finally, tobacco smoking is still a common and important risk factor for development of cardiovascular disease in teenagers and young adults [68]. In a questionnaire study of US adolescents with JIA, as many as 15.4 % reported use of tobacco in the last year [69]. A cross-sectional survey from Switzerland, of 7253 adolescents aged 16 to 20 years, adolescents with a chronic condition, defined as a disability or a disease lasting > 6 months and requiring continuous medical care, reported significantly higher rates of risk behavior including tobacco smoking than in a comparison group of healthy adolescents [70]. The investigation by Aulie et al. [37] likewise reported a significantly higher rate of daily smoking in the patients than in controls.

## Conclusions

In this review of the current literature we find convincing evidence for the existence of subclinical signs of premature atherosclerosis in patients with JIA, but the studies available do not provide a clear picture as to the cause. Inflammation is probably a driver, but attention must also be paid to other known risk factors for development of atherosclerosis, including obesity, physical inactivity and tobacco smoking - risk factors which are open to modification by changes in lifestyle.

With the advent of increasingly effective drugs for treating chronic inflammatory diseases in childhood and adolescence, and the resulting reduced risk of concomitant functional impairment, we should now broaden our strategy of management and address other potential consequences of chronic disease.

Reestablishment of a healthy lifestyle, including avoidance of adipositas and physical inactivity, is of great importance for gaining the full benefit of effective anti-inflammatory treatment and in securing a healthy life in adulthood.
